# Mothers' Willingness to Use Workplace Lactation Supports: Evidence from Formally Employed Mothers in Central Kenya

**DOI:** 10.1016/j.cdnut.2023.102032

**Published:** 2023-11-11

**Authors:** Scott B. Ickes, Hellen Sankaine Lemein, Anna McKay, Kelly Arensen, Benson Singa, Joyceline Kinyua, Ruth Nduati, Judd Walson, Donna M. Denno

**Affiliations:** 1 Department of Biological and Health Sciences, Wheaton College, Wheaton, IL, United States; 2 Department of Health Systems and Population Health, University of Washington, Seattle, WA, United States; 3 Departments of Medicine (Allergy and Infectious Disease) and Epidemiology, University of Washington, Washington, DC, United States; 4 Department of Kinesiology, William and Mary, Williamsburg, VA, United States; 5 Department of Pediatrics and Child Health, University of Nairobi, Nairobi, Kenya; 6 Department of Global Health, University of Washington, Seattle, WA, United States; 7 Department of Pediatrics, University of Washington, Seattle, WA, United States; 8 Department of International Health, Johns Hopkins Bloomberg School of Public Health, Baltimore, MD, United States

**Keywords:** workplace lactation supports, breastfeeding policy, maternal perceptions, breastmilk expression, infant and young child feeding

## Abstract

**Background:**

Formally employed mothers are vulnerable to early cessation of exclusive breastfeeding. Kenyan national policy requires employer-provided maternity benefits and workplace lactation supports.

**Objective:**

The objective of this study was to evaluate willingness to use nationally mandated workplace lactation supports among formally employed women in Kenya.

**Methods:**

We conducted a cross-sectional survey among 304 mothers of children ages ≤12 mo in Naivasha, Kenya, who were currently formally employed and employed before delivery of the most recent child to assess availability of and willingness to use current and potential future workplace lactation supports.

**Results:**

The most available reported workplace lactation supports were schedule flexibility to arrive late or leave early (87.8%) or visit a child to nurse during lunch (24.7%), followed by company-funded community-based daycare (7.6%). Few (<4.0%) reported the availability of lactation rooms, on-site daycares, transportation to breastfeed during lunch, refrigerators for expressed milk, or manual or electric breastmilk pumps. If made available, >80% of mothers reported moderate or strong willingness to use flexible schedules to arrive late or leave early, break during lunch, and transportation to visit a child to nurse. A moderate proportion reported strong willingness to use on-site daycares (63.8%), company-funded community-based daycare (56.9%), on-site lactation rooms (60.5%), refrigeration for expressed milk (49.3%), manual (40.5%), and electric pumps (27.6%). Mothers expressed fear of missing production targets and reported more willingness to use on-site compared with off-site daycare to save transportation time but noted concerns about chemical exposures and early arrival times with young infants. Hesitations regarding the use of on-site lactation rooms included concerns about privacy, milk identification and storage, and use and sharing of pumps.

**Conclusions:**

Flexible schedules were the workplace lactation supports in highest demand among formally employed mothers. Maternal willingness to use lactation rooms, refrigeration, and pumping equipment was moderate to low, suggesting sensitization may help to increase demand as the implementation of Kenyan policies moves forward.

## Introduction

Infants exclusively breastfed for the first 6 mo of life experience lower infection and mortality rates [1]. To support optimal growth, development, and to prevent child morbidity and mortality, infants are recommended to be breastfed within the first hour after birth, and to be breastfed exclusively through 6 mo, and to be continuously breastfed along with complementary foods for ≥2 y [2]. The effects of early cessation of exclusive breastfeeding (EBF) and suboptimal breastfeeding through the first year are more pronounced in low- and middle-income countries (LMICs), where the lack of sufficient infrastructure for clean water and sanitation increases risks of illness and mortality [3]. Although progress toward reducing under-5 mortality has been made in the past 2 decades, countries in sub-Saharan Africa experience the world’s highest under-5 mortality rates [2,4]. EBF trends and progress toward global targets have been variable across regions. Globally, fewer than half of children are breastfed exclusively for 6 mo [5]. Within Africa, there is a substantial regional variation in EBF rates: 55% of infants aged <6 mo are breastfed exclusively in Eastern and Southern Africa, compared with only 32% in North Africa and the Middle East [3,6], and has had considerable increases in rates of EBF [2], Most LMICs are off target to meet the World Health Assembly goal of EBF rates of 70% by 2025 [7].

Female workforce participation rates in sub-Saharan Africa have been relatively high and stable over the past 2 decades, with most recent estimates of just >60% engaged in paid work, compared with 47% worldwide (ILO, 2023). Kenya has one of the highest female workforce participation rates at 72% (ILO, 2023). Naivasha is one of the regions in Kenya that employs many women in formal commercial agriculture, which has been documented to pose added challenges to BF due to physical demands, working hours, and long commutes [19]. Maternal employment is an established barrier to EBF in many LMICs [8,9]).

Mothers engaged in all employment types face challenges in practicing EBF for the recommended duration. However, formally employed women receive more protections and support through national policies and benefits intended to support lactation after resuming paid work than informally employed women [4,10].

Only 63 (32.8%) of 192 countries surveyed in the Global Nutrition Report had national policies to promote EBF in infants aged <5 mo [11]. The most prevalent legal support for breastfeeding is mandatory maternity leave. According to the International Labour Standards Maternity Protection Convention (2000), mothers should receive a minimum of 14 wk—and optimally 18 wk—of paid maternity leave with a provision of no less than 2/3 of their previous earnings. At the time of the latest assessment, only 11% of countries have 18-wk paid maternity leave policies [4]. The International Labour Organization (ILO) further recommends comprehensive maternity protections, including pay and medical benefits during maternity leave, workplace health protection, employment protection and nondiscrimination, at least one daily breastfeeding break, and childcare facilities [12]. A 2014 review of breastfeeding-friendly workplace policies in 159 countries reported provisions in ≥121 countries for breaks or reduced daily working hours for nursing mothers [13]. When provided, nursing breaks are most commonly paid (114 countries). However, only 50 countries had national legislation regarding establishing sanitary facilities for nursing at or near the workplace [13].

Research on maternity protection has often focused on maternity leave, often ignoring other essential components of comprehensive support for working, lactating mothers [12]. There is limited evidence on maternal attitudes regarding available workplace lactation supports and the demand for new supports. This information is critically needed for evidence-informed policy development and implementation [14].

In Kenya, the 2017 Kenya Health Act, which is a national policy that requires employers to provide on-site lactation rooms and flexible work schedules in addition to a 3-mo paid maternity leave, is currently being implemented with variable compliance across all sectors. Formally employed mothers, especially those in commercial agricultural employment, are vulnerable to early cessation of EBF [6]. A Kenyan Implementation Framework for Securing a Breastfeeding-Friendly Environment at Workplaces aims to support creating more breastfeeding-conducive environments [15]. To guide the implementation of these recent policy initiatives and to benefit workplace lactation initiatives more generally, we surveyed employed mothers to identify available workplace lactation supports and mothers’ willingness to use these and other resources if made available.

## Methods

### Overview

Between June and August 2021, we conducted a cross-sectional survey among 300 mothers of children aged ≤12 mo who were currently formally employed and who were also employed before delivery of their most recent child to investigate the availability of workplace lactation supports and willingness to use the same and future supports if made available, in commercial horticultural farms in Naivasha, Kenya.

### Study setting

Located in Kenya’s Great Rift Valley, Naivasha has a population of ∼314,000 [16]. This area contains the largest concentration of commercial flower farms in the country, which serve as the primary source of employment in the region and employ ∼50,000 people, over half of whom are women [17]. Most commercial farm employees live in a few densely populated peri-urban informal settlements with inadequate sanitation services and limited access to electricity. In Naivasha, some commercial flower farms and other employers provide resources such as on-site childcare and employee housing. Childcare services utilized by employed mothers are mostly community-based, informal, and unregistered.

### Study participants and recruitment

The research team approached mothers of children aged <12 mo on the maternity ward or during immunization visits at the area’s 2 main public health care facilities: the Naivasha Sub-County Referral Hospital and the Karagita Dispensary, as well as strategic bus stations in town. The study recruitment took place between July 5 and August 17, 2021. Health facility staff introduced eligible mothers to the research team, all of whom were fluent in Swahili and members of the Naivasha community. Research staff recruited mothers Monday through Friday for all 6 wk of the study. The staff explained the study’s purpose and opportunity to participate to all mothers present in the immunization clinics and maternity wards on recruitment days. We explained the study definition of formal employment—receiving a regular wage and paying taxes—to all mothers, and only those who confirmed their current participation in this type of employment were eligible. Written, informed consent was obtained from all participants before beginning the survey. Surveys were administered verbally in Swahili, English, or other preferred language of the mother (e.g., Kikuyu, Maasai). Participants were provided 200 Kenyan Shillings ($2.00 USD) to show appreciation for their voluntary participation. The research team took multiple precautions to protect participants and researchers from COVID-19 infection, including providing free masks to all participants, access to hand sanitizer or handwashing facilities, and conducting the majority of interviews outdoors, or indoors with air circulation from open windows and physical distancing of 2 m.

### Data collection tools

The survey domains included the following: *1*) maternal demographics, *2*) maternal and child health status, *3*) employment history, *4*) awareness of current workplace lactation supports, and *5*) self-reported use of workplace lactation supports if currently available, or willingness to use if made available in the future. The survey is available online (Supplemental Table 1).

### Tool development and pilot testing

The survey was constructed based on multiple sources including the Demographic and Health Survey [18], a previous survey on maternal employment and breastfeeding [19] and prior studies of attitudes toward lactation support at workplaces [20]. The survey was pilot tested among 15 mothers >3 wk in the same settings as the eventual data collection. The 3-person research team (HS, AM, and SI) took notes during pilot testing about participant understanding of survey questions, clarity and dimensionality of responses, and overall comfort with the research process. The research team met 3 times to review and discuss the tool before the final tool was developed and translated into Kiswahili.

#### Maternal demographics, maternity care utilization, and child health status

Mothers reported if, in the past 2 wk, their child had symptoms or a diagnosis of any of the 5 common illnesses and symptoms: pneumonia, malaria, diarrhea, fever, and cough. Survey information included maternal demographics, healthcare access and utilization, employment history, awareness of current workplace lactation supports, and self-reported willingness to use the same and additional potential breastfeeding supports.

#### Type of employment

Formal employment was defined as having a contract and receiving a regular paycheck. Informal employment was defined as being periodically or irregularly employed. Self-employment was defined as being a business owner or one’s own boss. We assessed current employment types, as well as before the delivery of the most recent child. We also estimated the commuting distance to workplace and the method of commuting.

#### Availability and use of workplace lactation supports

We queried mothers about the availability and use of on-site childcare facilities, company-funded community-based childcare, flexible work schedules, transportation to childcare facilities, private lactation rooms at work, and milk expression and storage equipment. In addition to yes and no responses, open-ended responses were solicited for reasons for declining available supports.

#### Willingness to use supports

Participants responded to statements of willingness to use various workplace lactation supports if made available using a 5-response Likert scale: disagree strongly, disagree, neutral, agree, and strongly agree. We also queried mothers using open-ended questions to understand the reasons for their scalar responses.

### Data analysis

Descriptive statistics were calculated using STATA version 16.0. Open-ended responses were transcribed verbatim and thematic analysis was conducted in Qualtrics software. The research team developed the codebook through an inductive and deductive hybrid approach, involving a review of the interview guide and a close reading of initial transcripts [11]. Three team members (AM, HSL, and SBI) collaborated to double-code each transcript. We identified themes using a constant comparative method and a coding matrix, organized by code families [21]. The study adhered to the STROBE guidance (Supplemental Table 2) [22].

### Minimizing bias

The research team did not have prior knowledge of participants’ social or economic status during recruitment. The strategy to enroll mothers from both health care facilities and bus stops was developed to minimize the chance of selection bias by only recruiting at health care facilities or those who use company transportation. Our previous research indicates that these resources are used by most women employed in the commercial agriculture industry [6]. In addition, to mitigate reporting bias, when approaching women for participation, we did not promote the study as an employment study but rather as research to identify opportunities to support breastfeeding among working mothers, open to employed mothers in any employment sector.

### Ethics

All team members completed training on responsible conduct of research, survey procedures, and ethics. The Kiswahili translation of the survey was certified by fluent speakers at the Kenya Medical Research Institute (KEMRI) as part of the ethical review process. No minimum sample size was calculated because the data analysis plan did not include bivariate analysis. However, a target of 300 was used to generate a reasonably large sample to justify the 5 Likert survey options. The KEMRI Scientific Ethical Review Unit (protocol number 0112/3712) and the Wheaton College Institutional Review Board (protocol number 1150761-6) approved the study procedures.

## Results

Of 410 women approached, 340 mothers had children <12 mo, of whom 304 agreed to participate. The primary reasons cited for refusing to participate were lack of time (*n* = 27) and discomfort or lack of clarity with the study purpose (*n* = 9). Of those agreeing to participate, 191 were surveyed at the main public referral hospital, *n* = 10 at a public dispensary, and *n* = 103 at company bus stops. Of those interviewed at the hospital, approximately half were recruited from the maternity ward, and half were recruited from the immunization clinic.

Table 1 reports participant demographic characteristics. Most participants (82.9%) were employed in commercial flower or produce farms, representing 20 different agricultural employers, followed by the education sector (8.7%). The mean (SD) maternal age was 29.8 (5.1) y. Most participants were married (78.6%), and 62.1% completed some secondary education or higher. Most participants received 4 or more antenatal visits (83.9%), delivered in an institutional facility (99.3%), and were satisfied with the quality of care (94.7%). Communing distance to work varied with 2–5 km most prevalent although 33.2% indicated a commute of >10 km.

**TABLE 1:** Demographic and workplace data of cross-sectional survey sample (*n* = 304)

	Number	%
Tribe		
Kikuyu	116	52.5
Luyha	85	20.1
Luo	31	6.9
Kiisi	32	7.9
Kalenjin	6	2.6
Other	34	11.2
	304	100
Marital status		
Married	239	78.6
Single/divorced/separated/widowed	65	21.4
Maternal employment		
Formally employed	304	100
Type of employment		
Commercial agriculture/flower farm	252	82.9
Teachers	25	8.2
Secretarial/business/sales	8	2.6
Health care	8	2.6
Tourism/security	3	1.0
Other	8	2.6
Child morbidity in the 2 wk preceding the survey (fever, diarrhea, malaria pneumonia, and cough)	132	43.4
Maternal education		
None	1	0.3
Any primary to completed primary	114	37.5
Any secondary to completed secondary	122	40.1
Above secondary (some university, diploma, or completed university)	67	22.0
Maternal age (y)		
18–24	62	20.4
25–29	78	25.7
>30	157	51.6
Unknown	7	2.3
Distance to maternal workplace (km)		
≤1	26	8.6
2–5	125	41.1
6–10	52	17.1
>10	101	33.2
Health service and maternity factors		
Received 4 or more antenatal visits	255	83.9
Satisfied with quality of delivery care	288	94.7
Facility-based delivery (government or private hospital, dispensary or healthcare center)	302	99.3
High parity (4 or more live births)	47	15.5

Table 2 reports the availability and use of workplace lactation supports. The most commonly available support was a flexible work schedule allowing late arrival or early departure (87.8%). Less commonly available were flexible schedules allowing employees to visit a child during lunch to breastfeed (24.7%), company-funded community-based daycare (7.6%), workplace lactation rooms (3.6%), and on-site, company-funded daycare (3.3%). The remaining supports were reported as available to <3% of the sample: transportation to travel (to home or a childcare center) to breastfeed during lunch (2.3%), refrigerator for expressed milk storage (1.6%), manual breastmilk pump (1.0%), and electric breastmilk pump (0.7%).

**TABLE 2:** Availability and use of workplace lactation supports

	Availability of support			Use of support	
	Number	%	Number	%	Percent using of those with support available
Flexible schedule allowing late arrival or early departure	267	87.8	264	86.8	98.9
Flexible schedule allowing to visit child during lunch to nurse	75	24.7	67	22.0	89.3
Transportation to visit child for feeding during lunch break	7	2.3	6	2.0	85.7
On-site company-funded daycare	10	3.3	7	2.3	70.0
Company-funded daycare in the community (not on-site)	23	7.6	2	0.7	8.7
Lactation room^1^	11	3.6	8	2.6	72.7
Refrigerator for pumped milk	5	1.6	4	1.3	80.0
Manual pump to express breastmilk	3	1.0	1	0.3	33.3
Electric pump to express breastmilk	2	0.7	2	0.7	100.0

1 A lactation room is defined as a private space where women can breastfeed or pump and store milk. Although Kenyan national policy defines a lactation room as having a private space where women can breastfeed or pump and store milk in a refrigerator, we separated these 2 benefits and defined a lactation room as a “room to breastfeeding or express and store breastmilk” and asked about refrigerated storage in a separate question.

The vast majority of participants reported using available resources. When available, 98.9% of mothers took advantage of flexible work schedules to arrive late or leave early, 89.3% utilized lunch breaks to visit their child to breastfeed, 85.7% utilized employer-provided transportation, and 72.7% used workplace lactation rooms. Although on-site daycare, lactation rooms, and refrigeration for storing expressed breastmilk were not widely available, they were reportedly utilized by >70% of participants with access to these supports whereas company-funded community-based daycare was rarely utilized. Only 1 of 3 women with access to manual pumps reported using them whereas both participants who reported access to electric pumps reported utilization.

Willingness to use various lactation supports if made available is summarized in Table 3. Participants reported being most willing to use flexible arrive early/leave late schedules, break during lunch, and transportation to visit their child to nurse; >80% agreed or strongly agreed about their willingness to use these supports. A moderate proportion reported agreement or strong agreement with their willingness to use an on-site daycare (67.1%), company-funded community-based daycare (66.8%), on-site lactation rooms (65.1%), refrigeration for expressed milk (54.6%), manual pumps (52.3%), and electric pumps (35.2%). Mothers were most likely to indicate strong or moderate disagreement about being willing to use refrigeration (32.5%) and manual (32.6%) or electric (45.7%) breastmilk pumps.

**TABLE 3:** Mothers’ willingness to use breastfeeding supports if made available at their workplaces

“If my workplace provided a new policy such as…”	Strongly disagree	Disagree	Neutral	Agree	Strongly agree
An on-site daycare, I would be willing to use it.	22(7.2%)	62 (20.4%)	16 (5.3%)	10 (3.3%)	194 (63.8%)
A company-funded daycare in the community, I would be willing to use it.	22 (7.2%)	34 (11.2%)	45 (14.8%)	30 (9.9%)	173 (56.9%)
A flexible schedule that allows me to come late or leave early to nurse, I would be willing to use it.	1 (0.3%)	0 (0.0%)	8 (2.6%)	0 (0.0%)	295 (97.0%)
A flexible schedule that allows me to go to visit my child during lunch to nurse, I would be willing to use it.	7 (2.3%)	25 (8.2%)	21 (6.9%)	11 (3.6%)	240 (78.9%)
Transportation (by vehicle) to visit my child during my lunch break, I would be willing to use it.	9 (3.0%)	28 (9.2%)	22 (7.2%)	8 (2.6%)	237 (78.0%)
An on-site lactation room, I would be willing to use it.	22 (7.2%)	45 (14.8%)	39 (12.8%)	14 (4.6%)	184 (60.5%)
A refrigerator for pumped milk, I would be willing to use it.	36 (11.8%)	63 (20.7%)	39 (12.8%)	16 (5.3%)	150 (49.3%)
A manual pump to express breastmilk, I would be willing to use it.	42 (13.8%)	57 (18.8%)	46 (15.1%)	36(11.8%)	123 (40.5%)
An electric pump to express breastmilk, I would be willing to use it.	55 (18.1%)	84 (27.6%)	58 (19.1%)	23 (7.6%)	84 (27.6%)

### Theme 1: Concerns regarding work schedules

Mothers expressed hesitation to spend time away from work during the workday and having children at the workplace for fear of missing production targets. “I strongly disagree with using anything whatsoever provided by the company as good services because I have fears of them cutting my bonus. My salary might also reduce, and we hardly get enough to feed our families. The process of going back and forth would be time-consuming and stressful.” Many mothers expressed a preference for arriving late or leaving early to breastfeed at home, compared with using company transportation to breastfeed during lunch, which was perceived to take too long. Some mothers also believed working long hours would reduce their milk supply.

### Theme 2: Daycare preferences and distance to work

More participants were more willing to use on-site rather than off-site daycare due to saving transportation time, and they noted being able to breastfeed and keep the infant in closer proximity as comforting and convenient. “This will be very good because when I go to breastfeed during lunchtime, I [normally] walk for a long time. It is sweaty, and there is a lot of dust. I sometimes have to bathe, and sometimes I get back [to work] late.” Mothers who expressed preference for community-based daycare described fear of chemical exposure to children at the worksite (e.g., “There are a lot of chemicals so it will not be good to take a small kid to the farm.”) The impact of early arrival times and long travel distances for the children were other reasons for this preference: “The distance will make me not use it because [my] baby will be affected by cold; also, we use dangerous chemicals, and it’s far.”

### Theme 3: Privacy and hygiene

Participants shared concerns about privacy and expressed hesitation about sharing lactation rooms, pumping equipment, and refrigerated storage. One mother objected, “I prefer to breastfeed my baby in my private room, not one of many in number, and I would not want to pump either,” whereas another mother expressed, “I will use it if it’s clean and sterilized well to prevent various infections.” Participants were also fearful of how their milk would be handled after pumping and worried staff might confuse their milk with that of others.

### Theme 4: Reluctance to and fear of using pumping equipment

Of all the proposed workplace lactation supports, participants expressed the least willingness to use electric pumps to express breastmilk. Breastmilk pumps were a new concept to mothers in this setting and they expressed concerns about the potential harmful effects of pumping, especially with electric equipment. Participants’ reluctance was connected to unfamiliarity with, and fears of, using electric equipment. One mother expressed, “I am afraid of being electrocuted” whereas another shared, “I hear it can cause cancer.” Others noted hesitation to pump at work due to a lack of workplace refrigeration to store expressed milk.

## Discussion

Mothers are not the only responsible group to support breastfeeding. Improvements in breastfeeding practices require diverse interventions that engage multiple levels of the socioecologic model and include health systems, families, communities, and workplaces [23]. This study examined experiences with, and opinions about, various workplace lactation supports among a group of employed mothers from the Kenya’s Rift Valley region, where commercial agriculture particularly in the floriculture industry is the predominant source of employment for women [6]. Through a novel survey in a setting where policies have recently been passed to protect, promote, and support breastfeeding among employed mothers, we sought to identify maternal preferences for various employer-provided lactation supports and their rationales for these opinions.

The 2017 Kenya Health Act requires employers with over 50 employees to provide lactation rooms and flexible work schedules [24]. However, implementation has been slow [25], and hampered by the COVID-19 pandemic, financial barriers, and lack of technical support for implementation [19]. Mothers in our survey reported nationally mandated on-site lactation rooms and equipment and flexible schedules to nurse during the workday as infrequently available. Although most reported the availability and use of flexible arrival and departure schedules, fewer reported the option to visit a child to nurse during lunch breaks. And, although very few noted the availability of on-site lactation rooms, this resource was highly utilized among those with access. If provided, preference is reported for on-site over community-based daycare. However, even the possibility of on-site daycare was met with some hesitation due to concerns about early commuting times with children, fear about salary deductions to fund the care centers, and chemical exposure to children receiving care near farms. Participants were most hesitant about on-site lactation rooms and breast pumps for fear of chemical exposure and health effects, respectively. The concern over chemical exposure as a barrier to breastfeeding at work has limited documentation in the East African context. We have previously noted mother’s hesitations over expressing milk, for fear of contaminating milk expressed before bathing, and in close proximity to workplace chemicals [19]. In Naivasha, agricultural employers take several mitigation strategies to limit chemical exposure among lactation women, and workers in general, including restricted-entry intervals after spraying, required personal protective equipment, task-shifting spraying to male employees, and use of integrated pest management to reduce the need for chemical spraying. Despite these measures, anticipating the concerns of lactating mothers regarding chemical exposure at work is important for planning workplace breastfeeding promotion initiatives.

In the past decade, sub-Saharan African countries have developed maternity and lactation protection policies [26]. However, legislation on workplace lactation supports is still recent [27], and the exploration of willingness to use these workplace supports is limited.

A qualitative study in South Africa that was nested within a mentorship intervention to improve health care worker counseling on infant feeding examined maternal perspectives on breastfeeding education. Mothers in the intervention noted that health care workers rigidly focused on the importance of EBF while ignoring challenges upon return to work, and they did not actively prepare women to manage this transition [28]. This study, conducted in a context where employers are mandated to provide space and time for breastmilk expression or nursing, underscores the need to equip mothers with knowledge about the options and skills that may be available to enable sustained EBF after returning to work. Ideal counseling would additionally evaluate maternal readiness to use such supports, if available, and address fears or hesitations surrounding utilizing available workplace supports. A study conducted in the same context as the present study identified that mothers of infants under 6 mo who had access to on-site lactation rooms and visited their child in daycare located at or nearby workplaces was associated with a greater likelihood of EBF [29].

In a recent community readiness survey among tea plantation managers, health professionals, grandmothers, fathers, and lactating mothers employed in the tea plantations in Kericho, Kenya, the authors reported a high overall level of community readiness to support workplace lactation in the tea plantations. Among the scale domains, “leadership” had the highest readiness score, whereas knowledge of and availability of resources to support lactation had the lowest score [30]. The authors recommended that employers consider a “breastfeeding-friendly workplace intervention” through the provision of lactation rooms, breast pumps, breaks for lactating mothers, and flexible hours to increase maternal intention to continue breastfeeding after returning to work. They assumed that the availability of these resources would improve breastfeeding intentions but did not measure willingness or demand for such supports [30]. An evaluation study conducted in the same setting among commercial tea plantation workers who returned to work after a 3-mo maternity leave found that implementation of an infant-friendly workplace support package that exceeds supports that are legally mandated resulted in a significant increase in EBF, particularly beyond 3 mo, the age at which employer-supported maternity leave ends in Kenya [31]. The authors recommended that maintaining and enhancing EBF while working is more likely when employers provide supportive work environments that include lactation rooms, pumping and storage equipment, and quality childcare centers. Although the intervention’s impact suggests reasonable utilization of the supports, the study did not evaluate their perspectives on the desirability of different supports or conduct an impact pathway analysis to identify the most influential components [31].

A survey of urban working mothers of children aged <6 mo in Nairobi, Kenya, found that attitudes toward breastmilk expression were favorable, but only 41% of these mothers were expressing either regularly or occasionally, with pain, inadequate facilities, and lack of time cited as challenges [32]. Although the 2018 Baby-Friendly Hospital Initiative guidelines set a benchmark for ≥80% of mothers being able to correctly describe or demonstrate how to express milk, the proportion among these working mothers in Kenya was far below at only 34% [32]. Positive attitudes toward breastmilk expression among working mothers need to be further supported through education, lactation counseling, and improved workplace equipment and facilities.

The preferences of employed mothers for workplace lactation supports in this study correspond to evidence that various supports in other contexts can improve EBF practices. These include paid breastfeeding breaks [[33], [34], [35]], designated spaces for breastfeeding or expression [33,34,[36], [37], [38], [39], [40]], and flexible working hours [33,39,40]. Additional recommendations include on-site or nearby childcare [37,[41], [42], [43]] and counseling to help mothers manage breastfeeding after return to work, including specific support around milk expression [40,41,[44], [45], [46]].

Following the passage of the Kenya 2017 Health Act, to our knowledge, this is the first study to assess maternal attitudes and willingness to use workplace lactation supports in Kenya. Our study was strengthened by focusing on employed mothers in the commercial agriculture industry, a critical setting for supporting mothers vulnerable to early cessation of EBF, and with some additional representation of mothers across sectors. We have previously documented that maternity leave was widely available among formally employed mothers in this setting. Therefore, we focused the survey on supports that would support lactation beyond the 12-wk leave. Several factors limited our study. First, the study results are most applicable to the formalized employment sector. This employment sector, compared with informal or self-employment, such as domestic work or market trading, represents a unique and growing labor market where mothers may be provided higher wages than less-formal work but also face restrictions about bringing children to their workplaces [13,47]. Mothers in our study were most likely to be employed in low-wage commercial agriculture—a formal employment sector characterized by long work hours, lengthy commuting distances, and demanding physical labor [19]. Second, due to the study setting and desire to represent mothers in the commercial agriculture industry, the generalizability of findings to other types of employment may be limited. Third, because many mothers lacked access to workplace lactation support, our findings rely heavily on theoretical willingness to support. Although beneficial to know mothers’ perspectives regarding potential new resources, understanding maternal experiences with existing resources will benefit those implementing workplace lactation supports. Fourth, we did not assess infant feeding practices or reasons for breastfeeding cessation because this was the focus of a recent study in the same setting [6]. This limited the present study from correlating responses about willingness to use breastfeeding supports with current feeding practices.

This study examined the willingness of formally employed mothers to use workplace lactation supports. However, a large segment of the population outside the scope of this study is mothers who are informally or self-employed, who could account for as many as 9 out of 10 employed women in Africa [36,48] and who are likely to be more vulnerable to the impact of work throughout the lactation period compared with formally employed mothers [12,49]. We advise that similar methodologies are needed to better evaluate the lactation supports available to this population and their perceived needs to be able to attain higher rates of EBF through the first 6 mo. However, different strategies such as maternity benefits through cash transfer may be more feasible for this population [23]. Most mothers in the present study were employed in the commercial floriculture industry. Although these findings relate to other women employed in low-wage, manual labor, their generalizability may be limited to other sectors with higher wages, more flexible working hours, and shorter commuting times.

To better promote, protect, and support breastfeeding among employed mothers, better awareness of maternal attitudes toward the potential workplace lactation supports is urgently needed. Our study highlights that supporting breastfeeding at work may be possible through the continued support for flexible work schedules, the funding and formation of on-site daycares, and milk expression and storage opportunities at work. The latter supports may require additional sensitization and support to gain traction in workplaces, especially in settings where mothers face time constraints from production quotas, which may deter midday breaks, chemical exposure, or other hygiene concerns. Personal protective equipment and altered quotas for lactating mothers may address some of these concerns in commercial agriculture and related contexts. All of these strategies must pay close attention to the considerable physical, psychologic, and demands posed by breastfeeding, and recognize that the added work of breastfeeding must be respected and supported by institutions that recommend and endorse this healthful practice [50].

In conclusion, formally employed mothers of infants in Naivasha, Kenya, have access to some, but not all, federally-required workplace lactation supports. Maternal use of available lactation supports was high in this context. If made available, maternal willingness to utilize lactation rooms, refrigeration, and pumping equipment was moderate to low, suggesting that sensitization alongside implementation may be needed to promote demand. This information provides valuable input into the implementation process of workplace lactation policies and laws in other LMIC contexts.

We recommend the policy process and new initiatives to promote breastfeeding among employed mothers seek maternal input into intervention design, monitoring, and evaluations. Future research should consider mothers’ experiences with workplace lactation supports as these resources become more widely available across various employment sectors.

## Funding

SBI received support from NIH under award number 3K01TW010827-05S1.

## Data availability

Data described in the manuscript, code book, and analytic code will be made available upon request pending application and approval.

## Conflict of interest

The authors report no conflicts of interest.

## Author contributions

The authors’ responsibilities were as follows – SBI, DMD, RN, JW: designed research; SBI, HSL, AM, JK: conducted the research; HSL, SBI, AM: analyzed data or performed the data analysis; SBI, HSL, KA: wrote the paper; SBI: had primary responsibility for the final content; and all authors: read and approved the final manuscript.
