# Supplementary material for: Mothers' Willingness to Use Workplace Lactation Supports: Evidence from Formally Employed Mothers in Central Kenya
Source: Curr Dev Nutr. 2023 Nov 11;7(12):102032. doi: 10.1016/j.cdnut.2023.102032 (PMC10733674; doi:10.1016/j.cdnut.2023.102032)
Supplement: Multimedia component 1 [file mmc1.docx]

**Supplemental Table 1: Mother’s Willingness to Use Workplace Breastfeeding Supports Survey Tool**

**Vi00:INTERVIEW**

**ID #_______**

| **Demographic Information** | | |
| --- | --- | --- |
| vi01  vi02  vi03/  vi04  vi04b  vi05 | Basic information | Today’s date __ __/ __ __/ __ __ __ __  MM DD Y Y Y Y  Researcher’s ID (1=Hellen; 2= Scott; 3=Anna; 4=Hannah; 5=Other) __________________  Location of interview ____________  Contact (phone number) ____________  Residential Area  ____Karagita (1) ___ Kihoto (2) ___ Site (3) ____Town (4) ___Kabati (5) ___ Guest Inn (6) ___ Kanjo (7) ___ Other (8): please specify ________ |
| Nvsd01  vsd02 | Maternal age | What is your date of birth?  __ __ / __ __ / __ __ __ __  MM DD Y Y Y Y  What is your age? __ __ years  Mother’s Name: ________________________________ |
| vsd04 | Maternal background | What is your ethnicity?  __ Kikuyu (1) __ Kalenjin (2) __ Luyha (3) __ Luo (4) __ Pokot (5) __ Turkana (6)  __ Maasai (7) __ Kamba (8) __ Meru (9) __Kisii (10) __Other (11) __________ |
| vsd12 | Maternal education | What is your highest level of education?  __ None (0) __ Some primary (1) __ Completed primary (2) __ Some secondary (3)  __ Completed secondary (4) __ Some college (5) __ Completed higher education (6) |
| vsd13 | Marital status | What is your marital status?  __ Married (1) __ Single/never married (2) __ Divorced/Separated (3)  __ Widowed (4) |
| vsd22  vsd23  vmorb1  vmorb2  vmorb3  vmorb4  vmorb5  vmorb6 | Infant information | What is your most recent baby’s name? _____________________________  What day was your most recent baby born? __ __ / __ __ / __ __ __ __  MM DD YYYY  Date of delivery: __ Before March 2020 (1) __ March – Aug 2020 (2) __ Sep 2020 - Present (3)  During the past two weeks, has (NAME) had any of the following illnesses?  __ diarrhea (3 or more loose stools in a day) (0=No; 1=Yes)  __ pneumonia (0=No; 1=Yes)  __ fever (0=No; 1=Yes)  __ malaria (0=No; 1=Yes)  __ cough (0=No; 1=Yes)  __ none of the above (0=No; 1=Yes) |
| vsd27  vsd28 | Parity | How many times have you been pregnant? ________ pregnancies  How many live births have you had? ________ live births |
| vbeh08  vbeh9 | HIV Status | Do you know your HIV status? __ Yes (1) __ No (0)  If yes, can you share your HIV status? __Positive (1) __Negative (0) __ NA (skip) (99) |
| vh01  vh02  vh03  vh04  vh05 | Setting of delivery | Where did you deliver (NAME)?  __ Government hospital (1) __ Private hospital (2) __ Government health centre (3)  __ Private health centre (4) __ Clinic (5) __ Dispensary (6) __ Home (7) __ Other (8): Please specify: _____________  What was the name of the facility (from last question)?  __ Naivasha District (1) __ Karagita Dispensary (2) __ Southlake Medical (3) __ Other (4): Please specify________  Did Covid change the place where you delivered your baby?  __ Yes (1) __ No (0)  Were you satisfied with the quality of care you received during your delivery?  __ Yes (1) __ No (0) __ NA (skip) (99)  If no, was your dissatisfaction due to Covid?  __ Yes (1) __ No (0) __ NA (skip) (99) |
| vh06  vh07  vh08  vh09  vh10 | Number of antenatal visits | While pregnant with (NAME), did you see anyone for antenatal care?  __ Yes (1) __ No (0)  If yes, how many times did you receive antenatal care (maternity care before your delivery) during this pregnancy? If no, NA (skip). ________ visits  Where do you receive antenatal care (NAME)?  __ Government hospital (1) __ Private hospital (2) __ Government health centre (3)  __ Private health centre (4) __ Clinic (5) __ Dispensary (6) __ Other (7): Please specify: _____________ __ NA (skip) (99)  What was the name of the facility (from last question)?  __ Naivasha District (1) __ Karagita Dispensary (2) __ Southlake Medical (3) __ Other (4): Please specify________ ___ NA (skip) (99)  Did Covid change the place you received antenatal care?  __ Yes (1) __ No (0) __ NA (skip) (99) |
| Vh11  Vh12  Vh13 | Setting of post-natal care (immunizations) | Where do you receive postnatal care?  __ Government hospital (1) __ Private hospital (2) __ Government health centre (3)  __ Private health centre (4) __ Clinic (5) __ Dispensary (6)  __ Other (8): Please specify, _____________ ___ NA (skip) (99)  What was the name of the facility (from last question)?  __ Naivasha District (1) __ Karagita Dispensary (2) __ Southlake Medical (3) __ Other (4): Please specify________ ___ NA (skip) (99)  Did Covid change the place you received postnatal care?  __ Yes (1) __ No (0) ___ NA (skip) (99) |
| Vh14 | Covid | Have you received the Covid vaccine?  __ Yes (1) __ No (0) |
| **Employment** | | |
| Ve01  Ve02  Ve03  Ve04  Ve05  Ve06  Ve07  Ve08  Ve09  Ve10  Ve11  Ve12 |  | **Before Covid**, did you perform any work for income or other type of compensation?  __Yes (1) __ No (0)  What type of employment did you have?  __ Formal (receive a regular paycheck, pay taxes) (0) __ Informal (periodic, irregular, do not pay taxes) (1) __ Self-employed (i.e. shop keeper) (2) __ Not employed outside of home (3) __ Student (4) __ NA  During the **first few months of Covid**, did you perform any work for income or other type of compensation? __ Yes (1) __ No (0)  What type of employment did you have?  __ Formal (receive a regular paycheck, pay taxes) (0) __ Informal (periodic, irregular, do not pay taxes) (1) __ Self-employed (i.e. shop keeper) (2) __ Not employed outside of home (3) __ Student (4) __ NA  Do you **currently** perform any work for income or other type of compensation?  __ Yes (1) __ No (0)  What type of employment do you currently have?  __ Formal (receive a regular paycheck, pay taxes) (0) __ Informal (periodic, irregular, do not pay taxes) (1) __ Self-employed (i.e. shop keeper) (2) __ Not employed outside of home (3) __ Student (4) __ NA (99)  What is your current occupation? That is, the work you normally do for income.  __ Teacher/Education (1) __ Clerical/Secretary (2) __ Business/Sales (3)  __ Healthcare (including midwifery, nursing) (4) __ Agriculture (5)  __ Domestic worker (6) __ Hotel/tourist industry (7)  __ Guard (security personnel-soldier or Askari) (8)  __ Flower or Produce Farm (9) Name of the farm:  __ Other (10) _________________________________  __ NA (99)  Do you usually work throughout the year, or do you work seasonally, or only once in a while?  __ Throughout the year/full-time (1) __ Seasonally/Part of the year (2) __ Once in a while (3) __ NA (99)  Did your employment status (informal, formal, self, etc.) change due to the pandemic?  __ Yes (1) __ No (0) __ (99) NA  Did your income change due to the pandemic?  __ Yes (1) __ No (0) ___ (99) NA  Distance to work in km _________ (estimate to nearest kilometer)  Mode of transportation to work: __ walk (1) ___ Company vehicle (2) ___public transport (3) ____ Other (4): Please specify_______ |

**Mother’s Readiness/Willingness to Use Maternity Policies Survey**

For mothers currently, formally employed in commercial farms, hotels, schools, healthcare, and other formal businesses. Do not administer to self-employed or informally employed mothers

| Vml01  Vml02  Vml03  Vml04 | Maternity Leave Policies | Did your place of work allow you time off after the birth of (NAME)?  __ Yes (1) __ No (0) __ Do not know (98) __ NA (99)  If your place of work allowed you time off after the birth of (NAME), did you use this time off?  __ Yes (1) __ No (0) __ NA (99)  Was the time off paid or unpaid? ___ Paid (1) ____ Unpaid (0) __ NA (99)  If you used the allowed time off after the birth of (NAME), how long did you not work?  ________ days ________ weeks ________ months Convert to weeks: ________  __ NA (99) |
| --- | --- | --- |

***For the next section, I am going to ask you about different supports available at your workplace and if you use them. If you do not know if a support exists, please state “I don’t know.”***

| **Variable** | **Support** | **Is ______ available?** | **Do you use ___?** |
| --- | --- | --- | --- |
| Vw01a  V101b | On-site daycare | __Yes (1)  __ No (0)  __ Don’t know (98) | __ Yes (1)  __ No (0)  __ NA (99) |
| Vw02a  Vw02b | A company-funded daycare in the community (not on-site) | __Yes (1)  __ No (0)  __ Don’t know (98) | __ Yes (1)  __ No (0)  __ NA (99) |
| Vw03a  Vw03b | A flexible schedule that allows you to come late or leave early | __Yes (1)  __ No (0)  __ Don’t know (98) | __ Yes (1)  __ No (0)  __ NA (99) |
| Vw04a  Vw04b | A flexible schedule that allows you to go home ***during lunch*** to nurse | __Yes (1)  __ No (0)  __ Don’t know (98) | __ Yes (1)  __ No (0)  __ NA (99) |
| Vw05a  Vw05b | Transportation to visit your child during your lunch break **(*explicitly a vehicle)*** | __Yes (1)  __ No (0)  __ Don’t know (98) | __ Yes (1)  __ No (0)  __ NA (99) |
| Vw06a  Vw06b | A lactation room (a private space where women can breastfeed or pump and store milk) | __Yes (1)  __ No (0)  __ Don’t know (98) | __ Yes (1)  __ No (0)  __ NA (99) |
| Vw07a  Vw07b | A refrigerator for pumped milk | __Yes (1)  __ No (0)  __ Don’t know (98) | __ Yes (1)  __ No (0)  __ NA (99) |
| Vw08a  Vw08b | A manual pump to express breastmilk | __Yes (1)  __ No (0)  __ Don’t know (98) | __ Yes (1)  __ No (0)  __ NA (99) |
| Vw09a  Vw09b | An electric pump to express breastmilk | __Yes (1)  __ No (0)  __ Don’t know (98) | __ Yes (1)  __ No (0)  __ NA (99) |
| Vw10  Vw10b | Other. Please list. __________________________________________ | __Yes (1)  __ No (0)  __ Don’t know (98)  __ NA (99) | __ Yes (1)  __ No (0)  __ NA (99) |

Please describe your responses to the questions above, especially if a support is offered and you don’t use it. _________ __________________________________________________________________________________________________

***“Next, I am going to give several statements about how you would respond in case a new support was made available to you by your employer. I am going to read you a series of statements and you can tell me how much you agree or disagree with the statement. For supports that are currently available, please answer based on your willingness to use them now. Please ask if you do not understand a statement.”***

| Willingness to Use Workplace Supports | | | | | | |
| --- | --- | --- | --- | --- | --- | --- |
|  | *“****If my workplace provided a new policy such as…*** | Strongly Disagree | Disagree | Neutral | Agree | Strongly Agree |
| Vws01c | An on-site daycare, I would be willing to use it. | 1 | 2 | 3 | 4 | 5 |
| Vws02c | A company-funded daycare in the community, I would be willing to use it. | 1 | 2 | 3 | 4 | 5 |
| Vws03c | A flexible schedule that allows me to come late or leave early to nurse, I would be willing to use it. | 1 | 2 | 3 | 4 | 5 |
| Vws04c | A flexible schedule that allows me to go to visit my child during lunch to nurse, I would be willing to use it. | 1 | 2 | 3 | 4 | 5 |
| Vws05c | Transportation (a vehicle) to visit my child during my lunch break, I would be willing to use it. | 1 | 2 | 3 | 4 | 5 |
| Vws06c | A lactation room, I would be willing to use it. | 1 | 2 | 3 | 4 | **5** |
| Vws07c | A refrigerator for pumped milk, I would be willing to use it. | 1 | 2 | 3 | 4 | 5 |
| Vws08c | A manual pump to express breastmilk, I would be willing to use it. | 1 | 2 | 3 | 4 | 5 |
| Vws09c | An electric pump to express breastmilk, I would be willing to use it. | 1 | 2 | 3 | 4 | 5 |

Please describe your response to each of the above: ________________________________________________________

Open-ended conclusion. Is there anything else that I should know about supports that your workplace provides or your experiences with these supports?
